# Supplementary material for: Molecular Pathways Linking High-Fat Diet and PM2.5 Exposure to Metabolically Abnormal Obesity: A Systematic Review and Meta-Analysis
Source: Biomolecules. 2024 Dec 16;14(12):1607. doi: 10.3390/biom14121607 (PMC11674716; doi:10.3390/biom14121607)
Supplement: Supplementary file 1 [file biomolecules-14-01607-s001.zip › biomolecules-3350555-supplementary.pdf]

Supplementary material

Molecular Pathways Linking High-Fat Diet and PM2.5 Exposure to Metabolically Abnormal Obesity: A Systematic Review and Meta-Analysis

Table S1. Procedure for searching and collecting articles 1

Figure S1. Enrichment analysis of gene sets (GSEA) and over-representation analysis (ORA) 2

Table S1. Procedure for searching and collecting articles

DATABASE	SCOPUS			
**Search field**	**Word 1**	**Boolean operator**	**Word 2**	**Search results**
Article title, Abstract, Keywords	PM2.5	AND	“High-fat diet”	**45**
Article title	PM2.5	AND	“High-fat diet”	**9**
Abstract	PM2.5	AND	“High-fat diet”	**44**
Keywords	PM2.5	AND	“High-fat diet”	**8**
**DATABASE**	**PUBMED**			
Title	PM2.5	AND	“High-fat diet”	**0**
Title/Abstract	PM2.5	AND	“High-fat diet”	**11**
**DATABASE**	**WEB OF SCIENCE**			
Title	PM2.5	AND	“High-fat diet”	**7**
Topic	PM2.5	AND	“High-fat diet”	**53**
Abstract	PM2.5	AND	“High-fat diet”	**46**
Author Keywords	PM2.5	AND	“High-fat diet”	**3**

On July 14, the search of reference was conducted using the profile “PM2.5” AND “High-fat diet” in the fields “Title,” “Abstract,” “Keywords,” and “Topic” in databases Scopus, Web of Science, and PubMed. The references were exported to the reference manager ENDNOTE.

Figure S1. Enrichment analysis of gene sets (GSEA) and over-representation analysis (ORA)

Procedure

Fold change values of transcriptors affected by exposure :
- High fat diet (HFD)
- $PM_{2.5}$
- HFD + $PM_{2.5}$

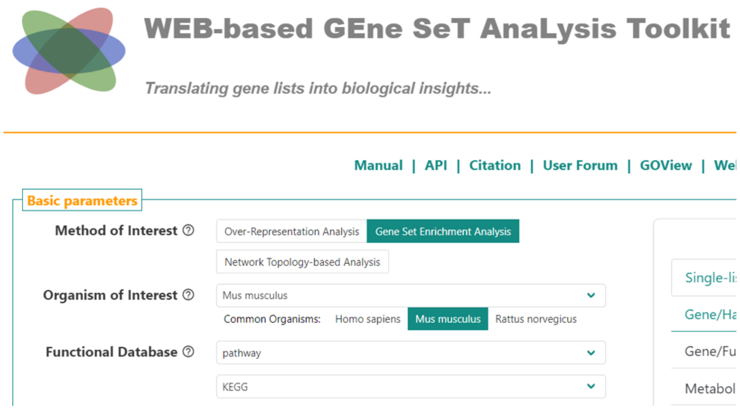

http://www.webgestalt.org/#

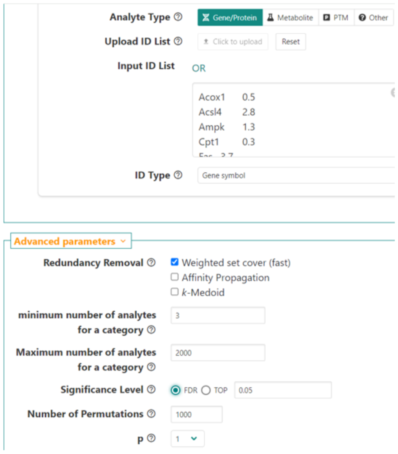

Selection criteria for enriched pathways GSEA:
- P-valor ≤ 0.05
- FDR: ≤ 0.05

Selection criteria for enriched pathwaysORA:
- P-valor ≤ 0.05
- FDR: ≤ 0.05
- Clústers with the highest number of transcripts

In both analysis, in addition the pathways corresponded to the tissue. 2

HDF exposure + filter air

Tissue	Statistically significant enrichment analysis
**Heart**	GSEA
**Liver**	ORA
**WAT**	GSEA
**BAT**	ORA

GSEA analysis of HFD-induced transcription in the heart, according to REACTOME

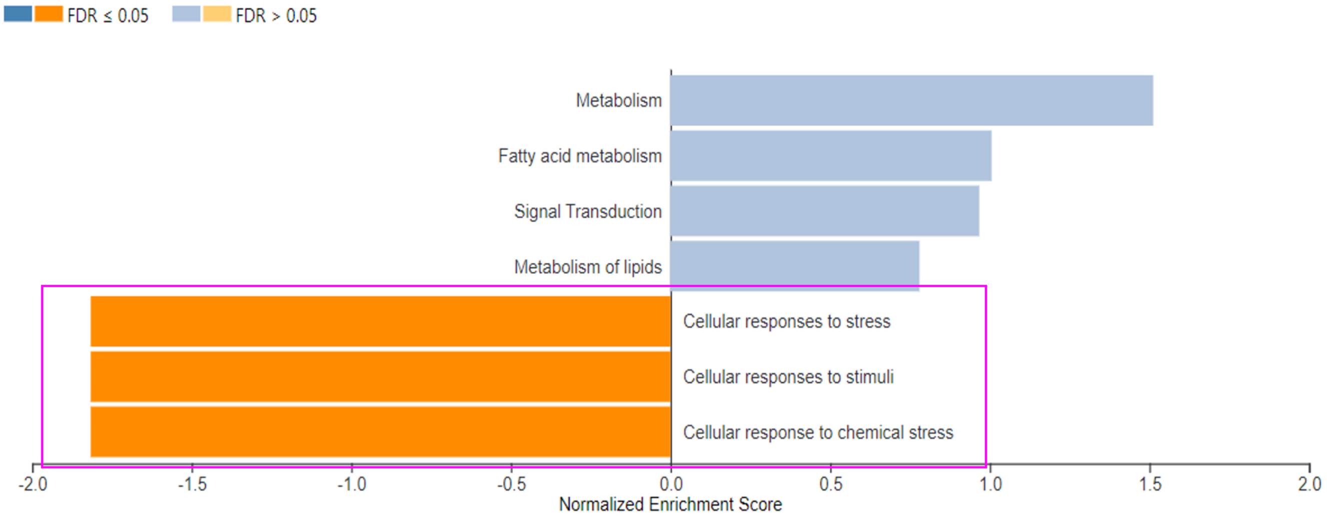

ID: R-MMU-2262752; Name: Cellular responses to stress
Size=3; Leading Edge Num=3; Normalized Enrichment Score=-1.82; P Value=2.469e-2; FDR=1.944e-2

User ID	Gene Symbol	Gene Name	Entrez Gene	Score
Gpx1	*Gpx1*	glutathione peroxidase 1	14775	0.95
Ppara	*Ppara*	peroxisome proliferator activated receptor alpha	19013	0.3
Sod1	*Sod1*	superoxide dismutase 1, soluble	20655	0.95

ID: R-MMU-8953897; Name: Cellular responses to stimuli
Size=3; Leading Edge Num=3; Normalized Enrichment Score= -1.82; P Value=2.469e-2; FDR=1.944e-2

User ID	Gene Symbol	Gene Name	Entrez Gene	Score
Gpx1	*Gpx1*	glutathione peroxidase 1	14775	0.95
Ppara	*Ppara*	peroxisome proliferator activated receptor alpha	19013	0.3
Sod1	*Sod1*	superoxide dismutase 1, soluble	20655	0.95

ID: R-MMU-9711123; Name: Cellular response to chemical stress
Size=3; Leading Edge Num=3; Normalized Enrichment Score=-1.82; P Value=2.469e-2; FDR=1.944e-2

User ID	Gene Symbol	Gene Name	Entrez Gene	Score
Gpx1	Gpx1	glutathione peroxidase 1	14775	0.95
Ppara	Ppara	peroxisome proliferator activated receptor alpha	19013	0.3
Sod1	Sod1	superoxide dismutase 1, soluble	20655	0.95

ORA analysis of HFD-induced transcription in the liver, according to KEGG

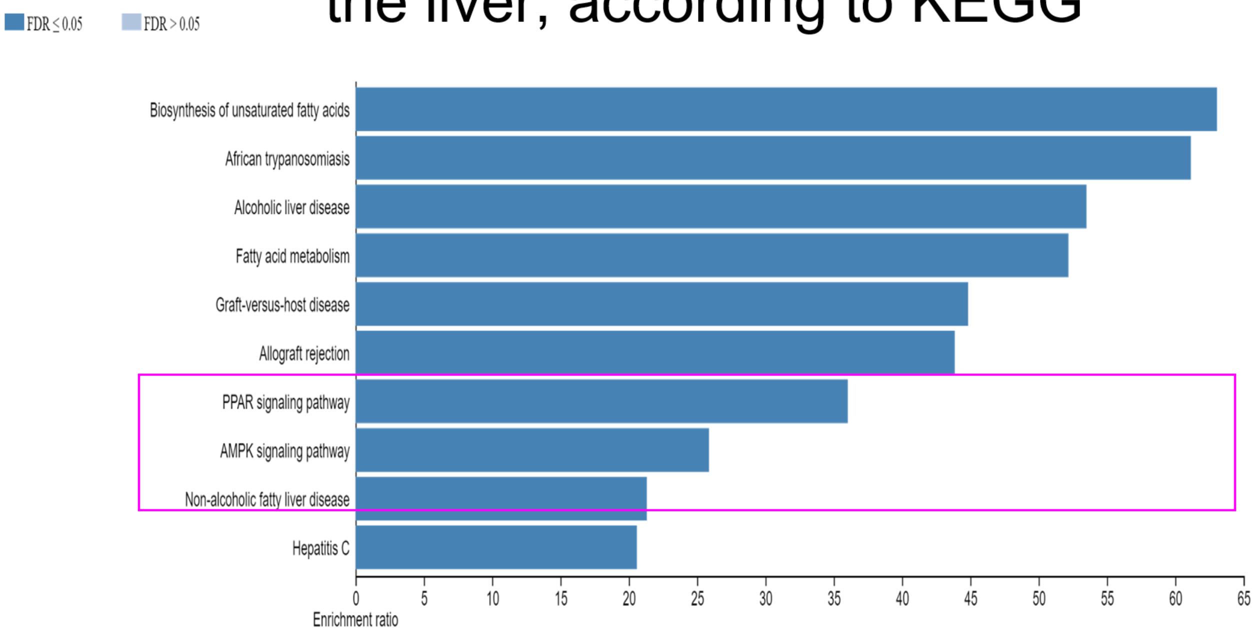

ID: mmu03320; Name: PPAR signaling pathway
size=84; overlap=3; expect=0.08; enrichmentRatio=36.03; PValue=5.868e-5; FDR=6.827e-3

User ID	Gene Symbol	Gene Name	Entrez Gene
Acox1	*Acox1*	acyl-Coenzyme A oxidase 1, palmitoyl	11430
Ppara	*Ppara*	peroxisome proliferator activated receptor alpha	19013
Scd1	*Scd1*	stearoyl-Coenzyme A desaturase 1	20249

ID: mmu04152; Name: AMPK signaling pathway
size=117; overlap=3; expect=0.12; enrichmentRatio=25.87; PValue=1.577e-4; FDR=1.376e-2

User ID	Gene Symbol	Gene Name	Entrez Gene
Fasn	Fasn	fatty acid synthase	14104
Scd1	Scd1	stearoyl-Coenzyme A desaturase 1	20249
Sirt1	Sirt1	sirtuin 1	93759

ID: mmu04932; Name: Non-alcoholic fatty liver disease
size=142; overlap=3; expect=0.14; enrichmentRatio=21.31; PValue=2.800e-4; FDR=1.804e-2

User ID	Gene Symbol	Gene Name	Entrez Gene
Fas	Fas	Fas cell surface death receptor	14102
Ppara	Ppara	peroxisome proliferator activated receptor alpha	19013
Tnf	Tnf	tumor necrosis factor	21926

GSEA analysis of HFD-induced transcription in the white adipose tissue (WAT), according to REACTOME

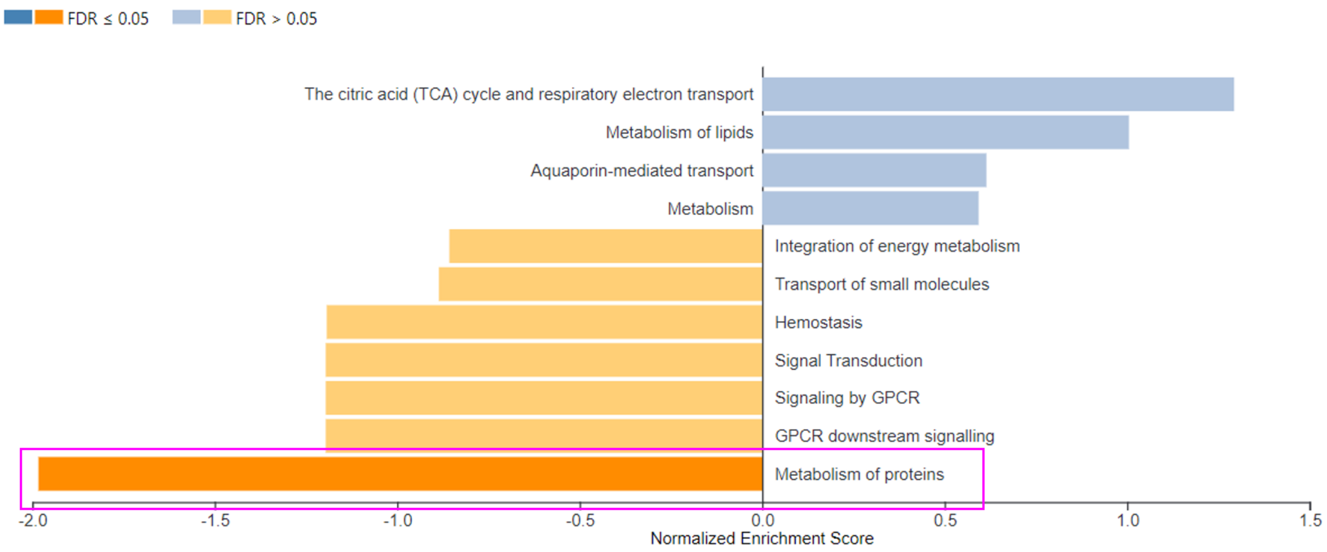

ID: R-MMU-392499; Name: Metabolism of proteins
Size=3
Leading Edge Num=3
Normalized Enrichment Score= -1.98
P Value=6.711e-3
FDR=2.495e-2

User ID	Gene Symbol	Gene Name	Entrez Gene	Score
Apoa1	Apoa1	apolipoprotein A-I	11806	0.6963
Apoa5	Apoa5	apolipoprotein A-V	66113	0.4808
Ghrl	Ghrl	ghrelin	58991	0.8129

ORA analysis of HFD-induced transcription in the brown adipose tissue (BAT), according to KEGG

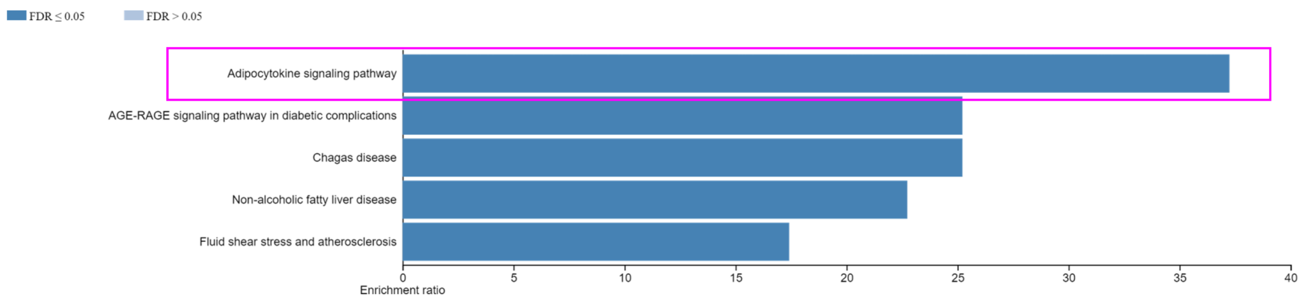

ID: mmu04920; Name: Adipocytokine signaling pathway
size=65; overlap=3; expect=0.08; enrichmentRatio=37.25; PValue=5.749e-5; FDR=1.003e-2

User ID	Gene Symbol	Gene Name	Entrez Gene
Lepr	*Lepr*	leptin receptor	16847
Ppara	*Ppara*	peroxisome proliferator activated receptor alpha	19013
Tnf	*Tnf*	tumor necrosis factor	21926

$PM_{2.5}$ exposure + normal diet

Tissue	Statistically significant enrichment analysis
**Heart**	GSEA
**Liver**	ORA
**WAT**	GSEA
**BAT**	ORA

GSEA analysis of $PM_{2.5}$-induced transcription in the heart, according to WikiPathway

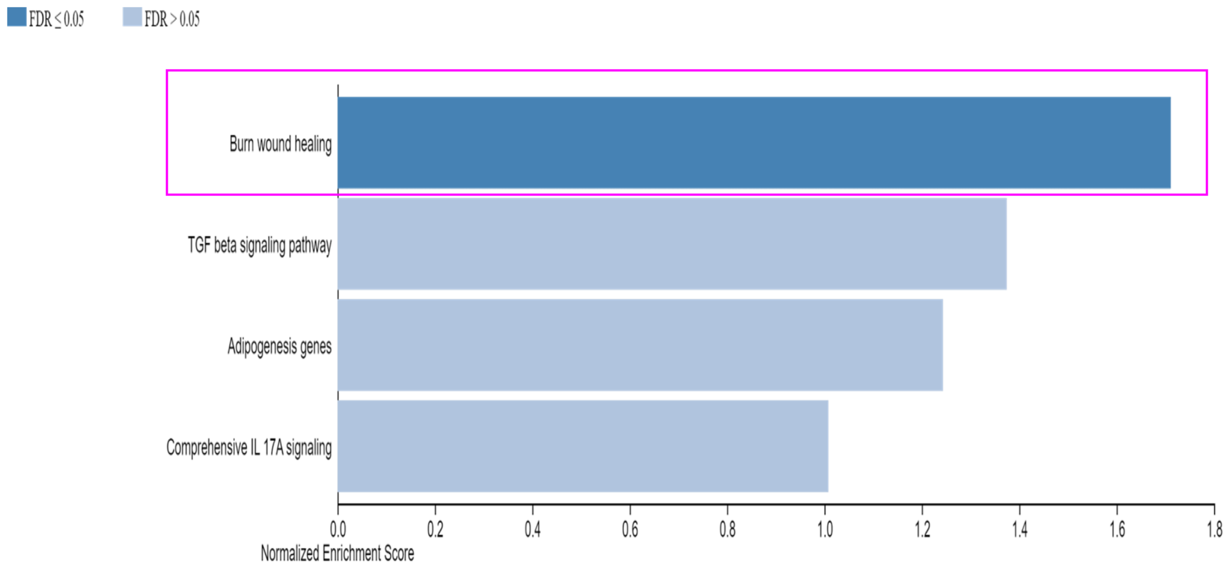

ID: WP5056; Name: Burn wound healing
Size=3; leadingEdgeNum=3; enrichmentScore=1.00; normalizedEnrichmentScore=1.71; PValue=9.917e-3; FDR=2.862e-2

User ID	Gene Symbol	Gene Name	Entrez Gene	Score
Col1a1	Col1a1	collagen, type I, alpha 1	12842	1.5
Tgfb1	Tgfb1	transforming growth factor, beta 1	21803	1.7
Tnf	Tnf	tumor necrosis factor	21926	3.9

ORA analysis of $PM_{2.5}$-induced transcription in the liver, according to KEGG

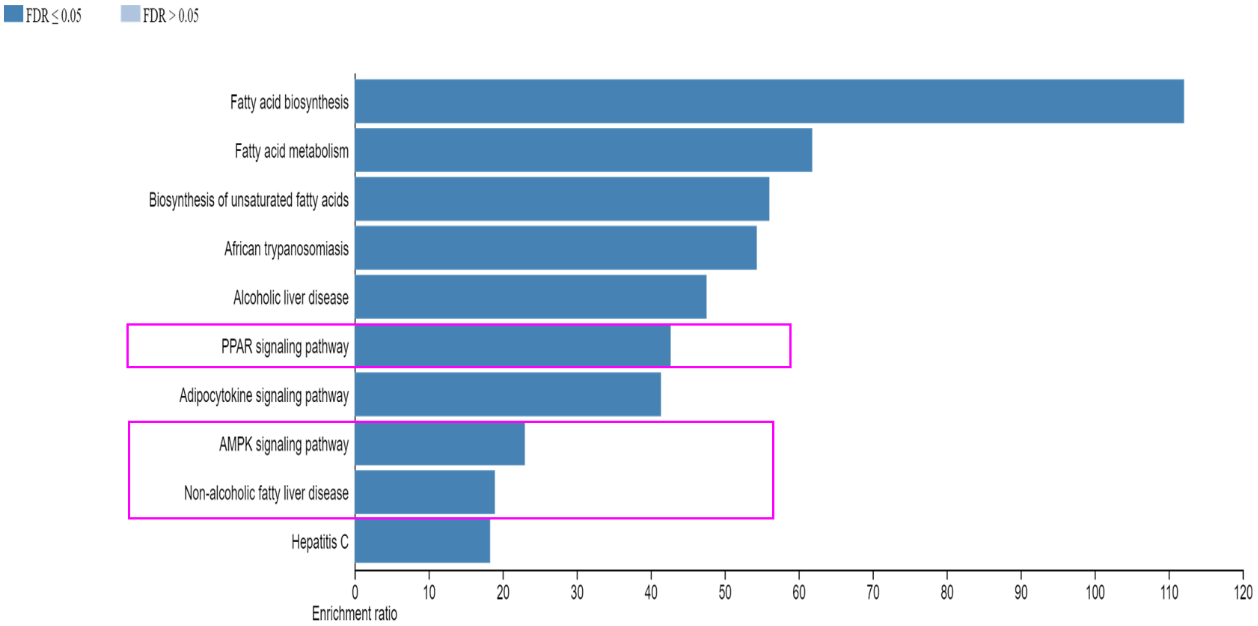

ID: mmu03320; Name: PPAR signaling pathway
size=84; overlap=4; expect=0.09; enrichmentRatio=42.70; PValue=1.323e-6; FDR=1.539e-4

User ID	Gene Symbol	Gene Name	Entrez Gene
Acox1	Acox1	acyl-Coenzyme A oxidase 1, palmitoyl	11430
Acsl4	Acsl4	acyl-CoA synthetase long-chain family member 4	50790
Ppara	Ppara	peroxisome proliferator activated receptor alpha	19013
Scd1	Scd1	stearoyl-Coenzyme A desaturase 1	20249

ID: mmu04152; Name: AMPK signaling pathway
size=117; overlap=3; expect=0.13; enrichmentRatio=22.99; PValue=2.341e-4; FDR=1.362e-2

User ID	Gene Symbol	Gene Name	Entrez Gene
Fasn	Fasn	fatty acid synthase	14104
Scd1	Scd1	stearoyl-Coenzyme A desaturase 1	20249
Sirt1	Sirt1	sirtuin 1	93759

ID: mmu04932; Name: Non-alcoholic fatty liver disease
size=142; overlap=3; expect=0.16; enrichmentRatio=18.94; PValue=4.146e-4; FDR=2.001e-2

User ID	Gene Symbol	Gene Name	Entrez Gene
Fas	Fas	Fas cell surface death receptor	14102
Ppara	Ppara	peroxisome proliferator activated receptor alpha	19013
Tnf	Tnf	tumor necrosis factor	21926

GSEA analysis of $PM_{2.5}$-induced transcription in the WAT, according to REACTOME

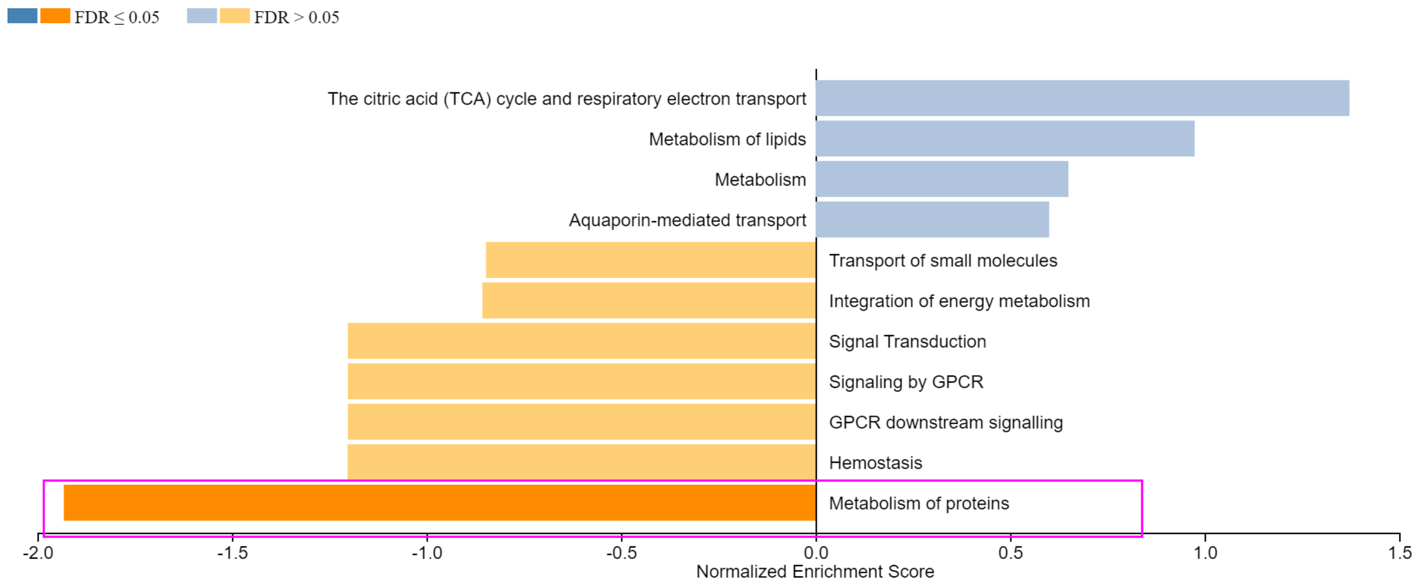

ID: R-MMU-392499; Name: Metabolism of proteins
Size=3
Leading Edge Num=3
Normalized Enrichment Score=-1.93
P Value=4.739e-3
FDR=4.824e-2

User ID	Gene Symbol	Gene Name	Entrez Gene	Score
Apoa1	Apoa1	apolipoprotein A-I	11806	0.4697
Apoa5	Apoa5	apolipoprotein A-V	66113	0.4575
Ghrl	Ghrl	ghrelin	58991	0.6605

ORA analysis of $PM_{2.5}$-induced transcription in the BAT, according to WikiPathway

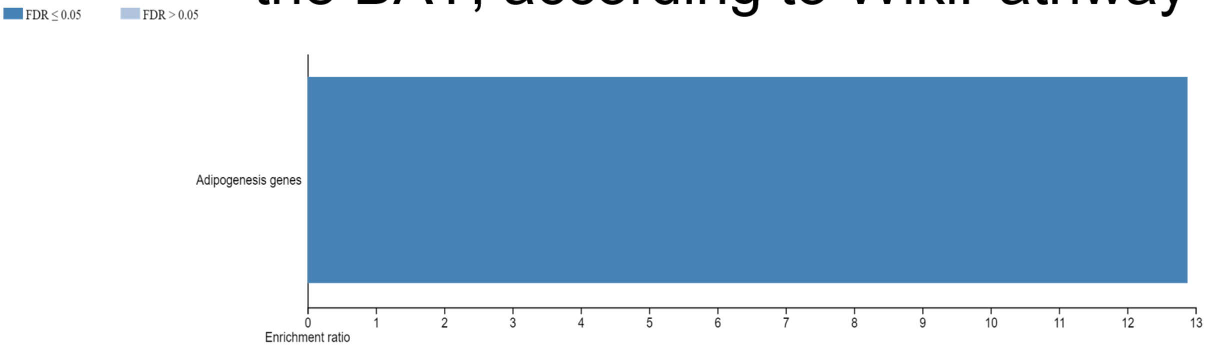

ID: WP447; Name: Adipogenesis genes

size=128; overlap=4; expect=0.31; enrichmentRatio=12.88; PValue=1.612e-4; FDR=3.030e-2

User ID	Gene Symbol	Gene Name	Entrez Gene
Cebpa	Cebpa	CCAAT/enhancer binding protein alpha	12606
Ppara	Ppara	peroxisome proliferator activated receptor alpha	19013
Serpine1	Serpine1	serine (or cysteine) peptidase inhibitor, clade E, member 1	18787
Ucp1	Ucp1	uncoupling protein 1 (mitochondrial, proton carrier)	22227

HFD + $PM_{2.5}$ exposure

Tissue	Statistically significant enrichment analysis
**Heart**	GSEA
**Liver**	ORA

GSEA analysis of HFD + $PM_{2.5}$-induced transcription in the heart, according to REACTOME

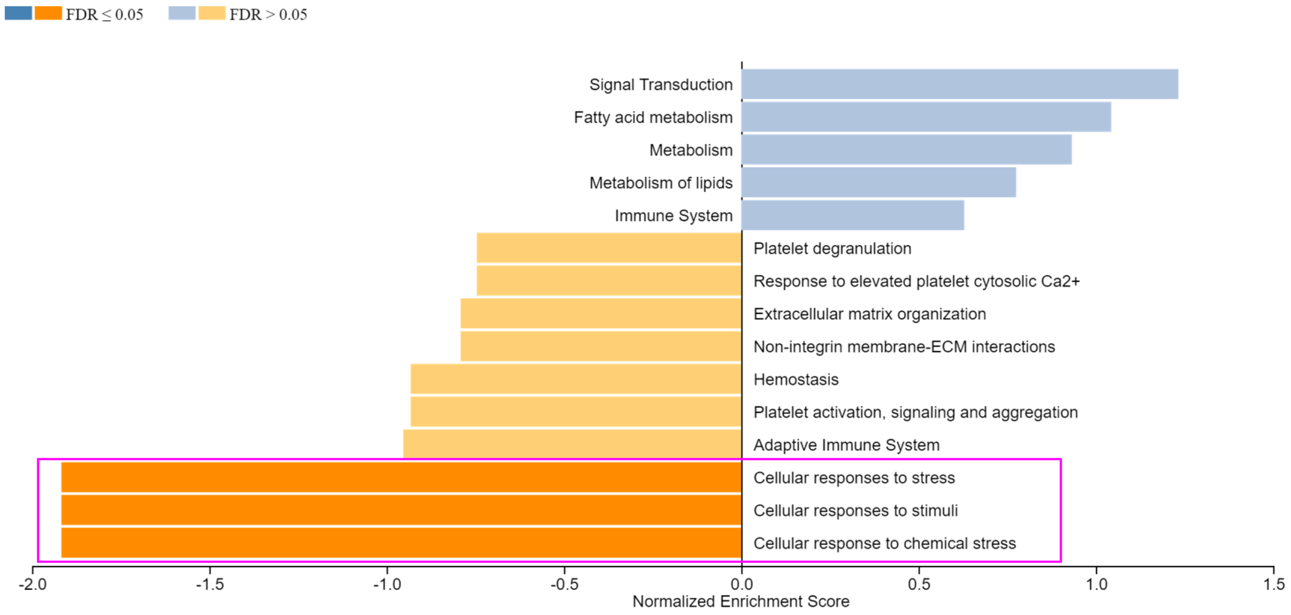

ID: R-MMU-2262752; Name: Cellular responses to stress
Size=3; leadingEdgeNum=3; enrichmentScore=-1.00; normalizedEnrichmentScore=-1.92; PValue=0.000e+0; FDR=2.274e-2

User ID	Gene Symbol	Gene Name	Entrez Gene	Score
Gpx1	Gpx1	glutathione peroxidase 1	14775	0.6
Ppara	Ppara	peroxisome proliferator activated receptor alpha	19013	0.9
Sod1	Sod1	superoxide dismutase 1, soluble	20655	0.45

ID: R-MMU-8953897; Name: Cellular responses to stimuli
Size=3; leadingEdgeNum=3; enrichmentScore=-1.00; normalizedEnrichmentScore=-1.92; PValue=0.000e+0; FDR=2.274e-2

User ID	Gene Symbol	Gene Name	Entrez Gene	Score
Gpx1	Gpx1	glutathione peroxidase 1	14775	0.6
Ppara	Ppara	peroxisome proliferator activated receptor alpha	19013	0.9
Sod1	Sod1	superoxide dismutase 1, soluble	20655	0.45

ID: R-MMU-9711123; Name: Cellular response to chemical stress
Size=3; leadingEdgeNum=3; enrichmentScore=-1.00; normalizedEnrichmentScore=-1.92; PValue=0.000e+0; FDR=2.274e-2

User ID	Gene Symbol	Gene Name	Entrez Gene	Score
Gpx1	Gpx1	glutathione peroxidase 1	14775	0.6
Ppara	Ppara	peroxisome proliferator activated receptor alpha	19013	0.9
Sod1	Sod1	superoxide dismutase 1, soluble	20655	0.45

ORA analysis of HFD + $PM_{2.5}$-induced transcription in the liver, according to KEGG

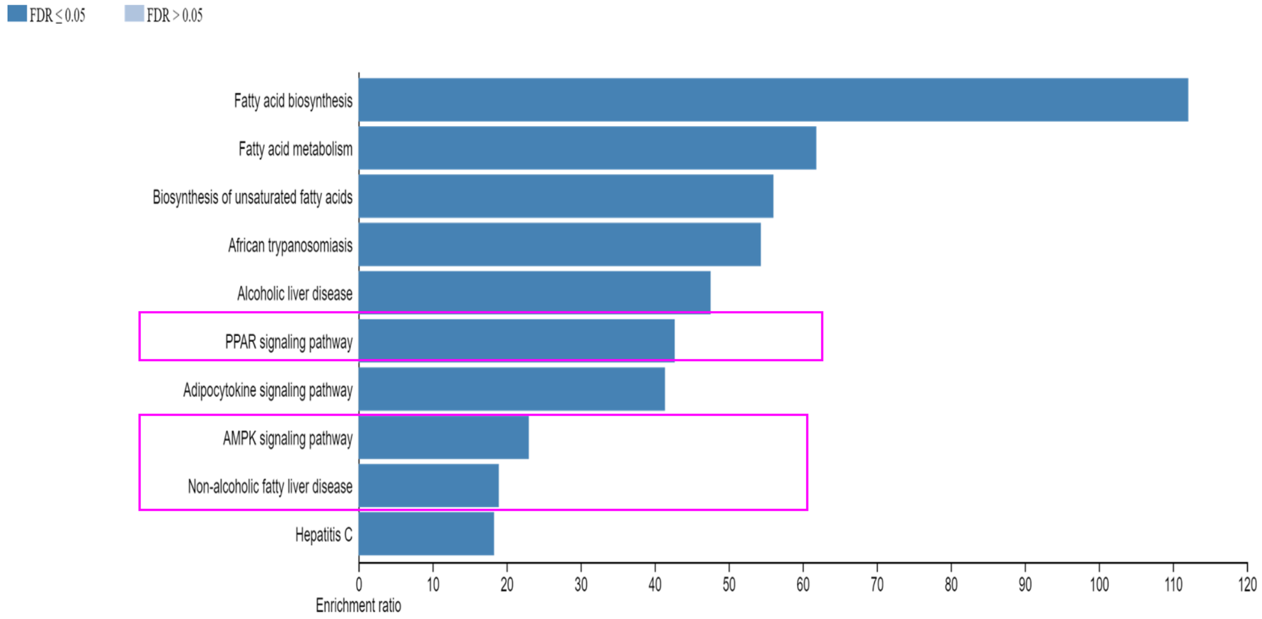

ID: mmu03320; Name: PPAR signaling pathway
size=84; overlap=4; expect=0.09; enrichmentRatio=42.70; PValue=1.323e-6; FDR=1.539e-4

User ID	Gene Symbol	Gene Name	Entrez Gene
Acox1	Acox1	acyl-Coenzyme A oxidase 1, palmitoyl	11430
Acsl4	Acsl4	acyl-CoA synthetase long-chain family member 4	50790
Ppara	Ppara	peroxisome proliferator activated receptor alpha	19013
Scd1	Scd1	stearoyl-Coenzyme A desaturase 1	20249

ID: mmu04152; Name: AMPK signaling pathway
size=117; overlap=3; expect=0.13; enrichmentRatio=22.99; PValue=2.341e-4; FDR=1.362e-2

User ID	Gene Symbol	Gene Name	Entrez Gene
Fasn	Fasn	fatty acid synthase	14104
Scd1	Scd1	stearoyl-Coenzyme A desaturase 1	20249
Sirt1	Sirt1	sirtuin 1	93759

ID: mmu04932; Name: Non-alcoholic fatty liver disease
size=142; overlap=3; expect=0.16; enrichmentRatio=18.94; PValue=4.146e-4; FDR=2.001e-2

User ID	Gene Symbol	Gene Name	Entrez Gene
Fas	Fas	Fas cell surface death receptor	14102
Ppara	Ppara	peroxisome proliferator activated receptor alpha	19013
Tnf	Tnf	tumor necrosis factor	21926
